# The Impact of Confounders on Symptom–Endoscopic Discordances in Crohn’s Disease

**DOI:** 10.1093/crocol/otad017

**Published:** 2023-03-28

**Authors:** Anjana Rajan, Yushan Pan, Prerna Mahtani, Rachel Niec, Randy Longman, Juliette Gerber, Dana Lukin, Ellen Scherl, Robert Battat

**Affiliations:** Division of Gastroenterology and Hepatology, New York—Presbyterian Hospital/Weill Cornell Medicine, New York, USA; Division of Gastroenterology and Hepatology, New York—Presbyterian Hospital/Weill Cornell Medicine, New York, USA; Division of Gastroenterology and Hepatology, New York—Presbyterian Hospital/Weill Cornell Medicine, New York, USA; Division of Gastroenterology and Hepatology, New York—Presbyterian Hospital/Weill Cornell Medicine, New York, USA; Division of Gastroenterology and Hepatology, New York—Presbyterian Hospital/Weill Cornell Medicine, New York, USA; Division of Gastroenterology and Hepatology, New York—Presbyterian Hospital/Weill Cornell Medicine, New York, USA; Division of Gastroenterology and Hepatology, New York—Presbyterian Hospital/Weill Cornell Medicine, New York, USA; Division of Gastroenterology and Hepatology, New York—Presbyterian Hospital/Weill Cornell Medicine, New York, USA; Division of Gastroenterology and Hepatology, New York—Presbyterian Hospital/Weill Cornell Medicine, New York, USA; Center for Clinical and Translational Research in Inflammatory Bowel Diseases, Centre Hospitalier de l’Université de Montréal, Montreal, Quebec, Canada

**Keywords:** Crohn’s disease, bile acid diarrhea, small intestinal bacterial overgrowth, inflammatory bowel disease, disease assessment

## Abstract

**Background:**

Discordances between clinical and endoscopic Crohn’s disease (CD) activity indices negatively impact the utility of clinic visits and efficacy assessments in clinical trials. Bile acid diarrhea (BAD) and small intestinal bacterial overgrowth (SIBO) mimic CD symptoms. This study quantified the impact of BAD and SIBO on the relationship between clinical and endoscopic disease activity indices.

**Methods:**

CD patients with 7α-hydroxy-4-cholesten-3-one (7C4) serum measurements and/or SIBO breath tests and matched clinical and endoscopic scores were included. Clinical remission (stool frequency [SF] ≤ 1 and abdominal pain score ≤ 1) rates were compared between those with and without (1) endoscopic remission, (2) BAD (7C4 > 55 ng/mL), and (3) SIBO.

**Results:**

Of 295 CD patients, 219 had SIBO testing and 87 had 7C4 testing. Patients with elevated 7C4 had lower proportions with clinical remission (14% vs 40%, *P* = .007) and SF ≤ 1 (14% vs 42%, *P* = .004) compared to those with normal 7C4. In patients with normal 7C4, higher rates of clinical remission (65% vs 27%, *P* = .01) and SF ≤ 1 (71% vs 27%, *P* = .003) existed in patients with endoscopic remission compared to those without endoscopic remission. Conversely, among the entire 295 patient cohorts, nearly identical clinical remission rates existed between those with and without endoscopic remission (25% vs 24%, *P* = .8), and the Crohn’s Disease Patient-Reported Outcome-2 score was not accurate for predicting endoscopic remission (Area Under the Curve (AUC): 0.48; 95% CI, 0.42–0.55). SIBO status did not impact clinical remission rates (*P* = 1.0).

**Conclusions:**

BAD, but not SIBO, contributed to symptom scores. A relationship between endoscopic inflammation and clinical remission rates only existed in patients without 7C4 elevations.

**Key Messages**

- **What is already known?**
- Clinical and endoscopic Crohn’s disease (CD) activity indices often do not align as symptoms are poorly representative of intestinal inflammation.
- **What is new here?**
- The absence of bile acid diarrhea (BAD) was associated with higher clinical remission rates measured with the Crohn’s Disease Patient-Reported Outcome-2 as well as greater concordance between clinical symptoms and endoscopic remission rates.
- **How can this study help patient care?**
- Testing for BAD as a symptom confounder can better inform clinicians on the reliability of symptom assessment as a surrogate for endoscopic inflammation.

## Introduction

Crohn’s disease (CD) is a chronic inflammatory bowel disease (IBD) that causes diarrhea and abdominal pain (AP). Symptom-based disease activity indices often guide therapeutic decisions.^1^ However, treating to achieve symptomatic remission has been demonstrated to be less important than achieving mucosal healing to impact patient outcomes in CD.^2^ Mucosal healing has been associated with prolonged remission and lower corticosteroid use, hospitalizations, and surgeries.^2–4^ For practical reasons, symptom-based scores are relied upon during office visits to assess disease activity. This is problematic as symptoms are poorly representative of intestinal inflammation, and there is frequent discordance between clinical and endoscopic disease activity indices.^5,6^ Importantly, there are no data on the impact of conditions that mimic CD symptoms, such as small intestinal bacterial overgrowth (SIBO) and bile acid diarrhea (BAD), on the relationship between symptoms and endoscopic inflammation.^7^ These entities may have impacted the results of clinical trials in CD patients, which routinely include those with likely high prevalence of BAD or SIBO, such as patients with a history of intestinal resection.^8^

It is imperative to understand how to accurately estimate intestinal inflammation using patient-reported symptoms when accounting for confounders commonly encountered. This is critical to timely management: During office-based visits, endoscopic evaluations are unavailable, noninvasive surrogates such as fecal calprotectin are not resulted at the time of the visit, and there is frequent nonadherence in returning samples.

Bile acid malabsorption causes a secretory diarrhea (BAD) that has increased prevalence in patients with ileal disease.^9^ In the United States, the 2 objective tests available for diagnosis include serum levels of 7-alpha-hydroxy-4-cholesten-3-one (7C4) and fecal bile acid measurement.^7^ 7C4 has been a validated method of diagnosing BAD in both IBS and IBD.^10–13^ SIBO is a condition in which the small bowel is overpopulated with colonic bacteria and has a variably reported prevalence of 25%–88% in CD.^14^ Symptoms of SIBO include diarrhea and AP which may mimic a CD exacerbation. The primary diagnostic tools used in practice are hydrogen and methane breath tests that are an indirect measure of bacterial metabolism of the substrate of choice (lactulose or glucose) in the small intestine.^15^ Lactulose breath tests are guidelines recommended as the preferred test, including for patients with prior intestinal surgery.^16^

No data exist on the impact of hypothesized frequent mimics of CD symptoms on symptom–endoscopic discordances. This study utilized SIBO and BAD results in a well-characterized cohort of CD patients to quantify the impact of both entities on the relationship between clinical and endoscopic disease activity indices in CD.

## Methods

### Patients and Study Design

Adult patients with CD at Weill Cornell Medicine/New York Presbyterian Hospital between May 2005 and November 2021 with an endoscopy within 75 days from a clinical score and either a SIBO lactulose or glucose hydrogen breath test or 7C4 serum test were included. SIBO tests were required to be within 180 days from the clinical score (mean: 45 days), and subgroups with SIBO testing within 30 days of prospectively collected clinical scores and without antibiotic use prior to clinical scores were identified (*n* = 80). Patients with BAD treatment within 2 weeks prior to either their 7C4 serum test or clinical score were excluded as were patients with IBD-related abdominal surgery between 7C4 testing and disease activity indices. The clinical score closest to the endoscopic score was preferentially selected. Available data on clinical scores, endoscopic scores, SIBO testing, and BAD testing along with demographic, medication, and surgical and medical history were collected through electronic medical records.

### Endpoints and Definitions

The primary aim was to evaluate the relationship between symptoms-based CD disease activity indices and (1) endoscopic inflammation, (2) BAD status, and (3) SIBO status. The CELEST trial assessed the efficacy of upadacitinib for CD.^17^ Despite meeting endoscopic remission (ER) endpoints, the trial failed to meet its co-primary endpoint of clinical remission (CR; stool frequency (SF) ≤ 1.5 and AP ≤ 1) but had more favorable results when exploring the modified clinical endpoint utilizing more liberal SF scores. Based on this, we hypothesized that SF played a major role in symptom–endoscopic discordances. In the present study, CR was defined as patients having both an SF ≤ 1 and AP ≤ 1 (hereafter referred to as CR 1/1), as this endpoint has been difficult to achieve in CD trials. In addition, individual components of SF and AP were analyzed separately to assess the relationships of individual components to endoscopic findings, BAD, or SIBO. ER was defined by Simple Endoscopic Score for CD (SES-CD) <3, Rutgeerts score <i2, or absence of ulceration.

The presence of SIBO was defined as a rise of hydrogen gas >20 parts per million (ppm) above baseline within 90 minutes of ingesting the substrate or a methane gas level >10 ppm at any point during the test. Substrates included lactulose (*n* = 217) and glucose (*n* = 2). The presence of BAD was defined as a serum 7C4 value >55 ng/mL based on the reference ranges provided (Prometheus Laboratories).

### Statistical Analysis

Data analysis included descriptive statistics computed for continuous variables (mean ± SD). Percentages were used for categorical variables. Between-group comparisons were performed using chi-square test, Fisher exact test, *t*-test, or Wilcoxon rank testing, as appropriate. The primary aim compared CR rates among those with and without (1) ER (2), BAD, and (3) SIBO. To assess the diagnostic accuracy of clinical scores for ER, area under the receiver-operating characteristic curve analyses assessed the diagnostic accuracy of Crohn’s Disease Patient-Reported Outcome-2 (CD-PRO-2, a continuous variable) for ER. A *P*-value of <.05 was considered significant. All analyses were done using STATA SE 15.1 (StataCorp).

## Results

### Patients

A total of 295 CD patients with matched clinical and endoscopic scores were included. Of these, 219 patients additionally had SIBO breath testing and 87 additionally had 7C4 testing available. Among those with SIBO tests, 74% were female, the mean age was 41 years old, 64% had nonstricturing, nonpenetrating disease, 28% had a history of surgical resection (73% of which was ileal or ileocolic), 50% were in ER, and 52% had positive SIBO results (Table 1). Of the SIBO-positive patients (*n* = 114), 58% were hydrogen gas (H_2_) positive only, 13% were methane gas (CH_4_) positive only, and 26% were both H_2_ and CH_4_ positive. Among patients with 7C4 testing, 39% were female, the mean age was 43 years old, 38% were in ER and 43% had elevated 7C4, indicating the presence of BAD (Table 1). Among patients with 7C4 testing, 66% had a history of intestinal resection (88% of which was ileal or ileocolic). The mean 7C4 concentration was 88.2 ± 112.2 ng/mL among all patients with measurements and 174.1 ± 129.0 ng/mL among 7C4 positive patients. Eleven patients had both SIBO and BAD testing completed and were included in respective analyses.

**Table 1.** Demographic and disease characteristics of total cohort and stratified by small intestinal bacterial overgrowth (SIBO) and bile acid diarrhea (BAD) result.

	Meets all criteria (*n* = 295)	SIBO (+) (*n* = 114)	SIBO (−) (*n* = 105)	BAD (+) (*n* = 37)	BAD (−) (*n* = 50)
Sex, female—*n* (%)	189 (64%)	84 (74%)	77 (73%)	16 (43%)	18 (36%)
Age in years—mean ± SD	41 ± 16	41 ± 15	41 ± 15	45 ± 18	42 ± 19
Race—*n* (%)					
White	198 (67%)	83 (73%)	72 (69%)	27 (73%)	25 (50%)
Black/African American	11 (4%)	1 (1%)	3 (3%)	1 (3%)	6 (12%)
Asian	7 (3%)	1 (1%)	3 (3%)	0	3 (6%)
Hispanic	8 (3%)	2 (2%)	2 (2%)	2 (5%)	2 (4%)
Other	21 (7%)	7 (6%)	9 (9%)	3 (8%)	3 (6%)
Unknown	50 (17%)	20 (18%)	16 (15%)	4 (11%)	11 (22%)
Montreal classification					
Age at dx—*n* (%)					
A1: ≤16	66 (22%)	31 (27%)	15 (14%)	12 (32%)	12 (24%)
A2: 17–40	169 (57%)	58 (51%)	68 (65%)	20 (54%)	27 (54%)
A3: >40	60 (20%)	25 (22%)	22 (21%)	5 (14%)	11 (22%)
Location—*n* (%)					
L1: Ileal	94 (32%)	42 (37%)	30 (29%)	14 (38%)	11 (22%)
L2: Colonic	50 (17%)	17 (15%)	27 (26%)	1 (3%)	5 (10%)
L3: Ileocolonic	145 (49%)	54 (47%)	45 (43%)	20 (54%)	33 (66%)
L4: Upper GI	6 (2%)	1 (1%)	3 (3%)	2 (5%)	1 (2%)
Behavior—*n* (%)					
B1: Inflammatory	159 (54%)	76 (67%)	64 (61%)	6 (16%)	16 (32%)
B2: Stricturing	63 (21%)	19 (17%)	20 (19%)	16 (43%)	15 (30%)
B3: Penetrating	73 (25%)	19 (17%)	21 (20%)	15 (41%)	19 (38%)
Perianal involvement—*n* (%)	78 (26%)	27 (24%)	21 (20%)	18 (49%)	16 (32%)
Previous resection—*n* (%)	111 (38%)	38 (33%)	24 (23%)	29 (78%)	28 (56%)
Ileocolic resection—*n* (%)	88 (30%)	27 (24%)	18 (17%)	28 (76%)	22 (44%)
Endoscopic remission—*n* (%)	135 (46%)	57 (50%)	52 (50%)	16 (43%)	17 (34%)
Medication at time of clinical score—*n* (%)					
5-ASAs	93 (32%)	36 (32%)	42 (40%)	6 (16%)	14 (28%)
Immunomodulators	52 (18%)	20 (18%)	21 (20%)	5 (14%)	8 (16%)
Steroids	48 (16%)	13 (11%)	17 (16%)	11 (30%)	10 (20%)
Biologics	116 (39%)	41 (36%)	28 (27%)	24 (65%)	32 (64%)

Abbreviations: 5-ASAs, 5-aminosalicylic acids; BAD, bile acid diarrhea; dx, diagnosis; GI, gastrointestinal; SD, standard deviation; SIBO, small intestinal bacterial overgrowth.

### Relationship Between Clinical and Endoscopic Disease Activity Indices

**Figure 1.**
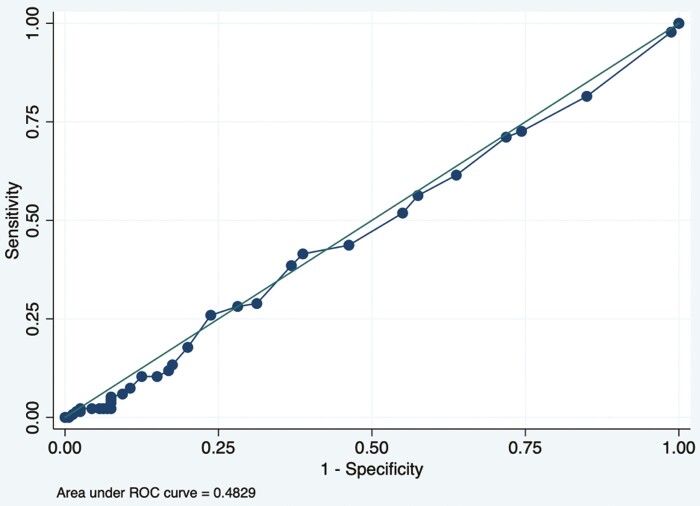
Crohn’s Disease Patient-Reported Outcome (CD-PRO)-2 score receiver-operating characteristic (ROC) curve in predicting endoscopic remission.

Among the 295 patients analyzed, the mean time between clinical and endoscopic scores was 21 ± 17 days. Among all patients, similar rates of CR 1/1 existed among patients with ER compared to those without ER (25% [34/135] vs 24% [38/160], *P* = .8). When assessing the individual components of CR, similar proportions of CR SF ≤ 1 existed among patients with ER versus without ER (36% vs 28%, *P* = .1). Proportions of CR AP ≤ 1 were not significantly different among patients with ER compared with those without ER (73% vs 83%, *P* = .06). CD-PRO-2 clinical score was not accurate in predicting endoscopic activity based on the receiver-operating characteristic curve (AUC: 0.48; 95% CI, 0.42–0.55, Figure 1).

### Relationship Between Bile Acid Diarrhea, Endoscopic Inflammation, and Symptom Scores

**Figure 2.**
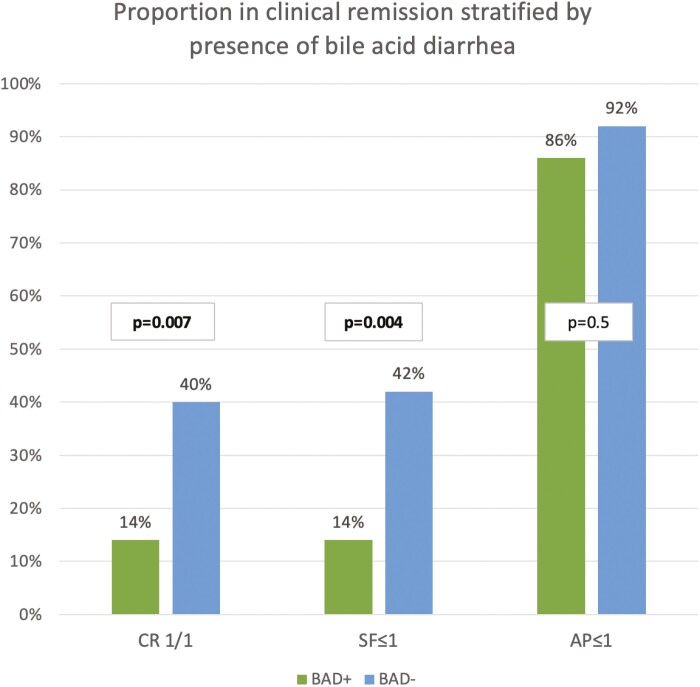
Proportion of patients in clinical remission stratified by presence of bile acid diarrhea. CR, clinical remission; 1/1, stool frequency (SF) ≤1 and abdominal pain (AP) ≤1; BAD+, bile acid diarrhea positive with 7C4 > 55 ng/mL; BAD−, bile acid diarrhea negative with 7C4 < 55 ng/mL.

Patients with elevated 7C4 had a significantly lower proportion of patients in CR 1/1 compared to patients with normal 7C4 (14% [5/37] vs 40% [20/50], *P* = .007; Figure 2). When analyzing subcomponents of clinical scores, patients with elevated 7C4 had lower proportions with SF ≤ 1 compared to those with normal 7C4 (14% vs 42%, *P* = .004; Figure 2). However, similar rates of patients had AP ≤ 1 when comparing those with and without elevated 7C4 (86% vs 92%, *P* = .5); Figure 2). In a subgroup analysis of patients with ileocolic resections, relationships between 7C4 elevations and CR yielded similar numerical differences to the overall population. Those with elevated 7C4 had numerically lower CR (CR 1/1) rates than those with normal 7C4 concentrations (14% [4/28] vs 36% [8/22], *P* = .07). Similarly, in a smaller subgroup of unoperated patients, greater numerical differences existed, with lower rates of CR 1/1 in those with elevated 7C4 compared to those with normal 7C4 (11% [1/9] vs 43% [12/28], *P* = .09).

**Figure 3.**
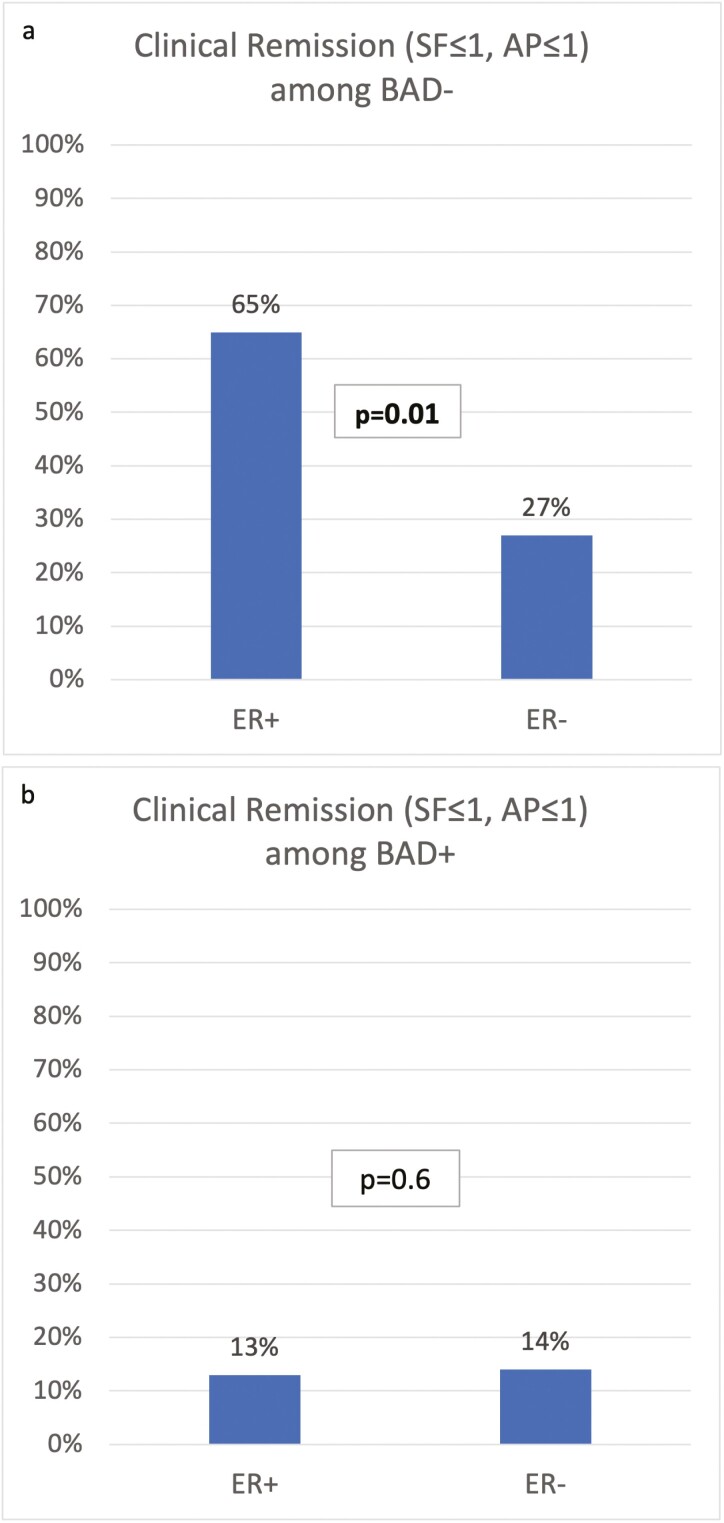
Proportion of patients in clinical remission stratified by endoscopic evidence of disease in (A) patients without bile acid diarrhea and (B) patients with bile acid diarrhea. BAD−, bile acid diarrhea negative with 7C4 < 55 ng/mL; BAD+, bile acid diarrhea positive with 7C4 > 55 ng/mL; ER+, endoscopic remission; ER−, endoscopic inflammation.

In patients with normal 7C4, higher rates of CR 1/1 (65% [11/17] vs 27% [9/33], *P* = .01; Figure 3A) and SF ≤ 1 (71% vs 27%, *P* = .003) existed in patients with ER compared to patients without ER. In addition, the rates of patients with AP ≤ 1 were similar in those with and without ER (94% vs 91%, *P* = 1.0) among this subgroup.

In patients with elevated 7C4, similar CR 1/1 (13% [2/16] vs 14% [3/21], *P* = 1.0) rates existed when comparing those with and without ER (Figure 3B).

### Relationship Between Small Intestinal Bacterial Overgrowth, Endoscopic Inflammation, and Symptom Scores

 In those with available SIBO breath testing, the mean time between SIBO testing and clinical scores was 45 ± 45 days. Patients with SIBO had identical CR 1/1 rates to those without SIBO (22% [25/114] vs 22% [23/105], *P* = 1.0). When assessing individual clinical disease activity components, SIBO-positive and -negative patients had identical proportions of patients with AP ≤ 1 (74% vs 74%, *P* = .9) or SF ≤ 1 (31% vs 31%, *P* = .9).

Among patients with negative SIBO breath test (*n* = 105), there was still no significant relationship between clinical and endoscopic disease activity indices. In these patients, similar rates of CR 1/1 existed among patients with ER compared to those without ER (19% [10/52] vs 25% [13/53], *P* = .5). Identical conclusions existed in patients with positive SIBO tests.

Analyses in patients with clinical scores that were prospectively collected, within 30 days of SIBO test and without antibiotics prior to clinical scores (*n* = 80) demonstrated that SIBO status (16% [6/38] SIBO+ vs 17% [7/42] SIBO−, *P* = .9) was not associated with CR 1/1. SIBO status was also not associated with SF ≤ 1 (29% SIBO+ vs 21% SIBO−, *P* = .4) or AP ≤ 1 (66% SIBO+ vs 69% SIBO−, *P* = .8).

## Discussion

CD often involves the ileum and ileocecal valve, potentially predisposing these patients to conditions such as SIBO and BAD that are thought potentially confound CD symptom–based disease activity indices. This study is the first to evaluate the impact of these alternative sources of symptoms, on CD activity assessments. Utilizing a well-characterized cohort with matched clinical and endoscopic assessments, this study presents a large cohort reinforcing the poor relationship between clinical disease activity scores and endoscopic inflammation.^5,6^ Uniquely, this study is also the first to formally quantify the reason for this discordance in CD and found that BAD, but not SIBO, significantly impacts CR rates. This study also found that the absence of BAD was associated with a reconciled relationship between endoscopic inflammation and patient symptoms.

Resection of the ileocecal valve increases the likelihood of colonic bacterial translocation upstream, and ileitis and/or ileal resection inhibits bile acid absorption. Our cohorts had a SIBO prevalence of 52% and a BAD prevalence of 43%, consistent with previously published data reinforcing the frequent coexistence of these conditions.^14,18^

Our findings showed that patients without BAD were more likely to be in CR compared to those with BAD. Notably, when isolating the relationship between SF and AP subcomponents, BAD only contributed to increased SF, not AP. This is a valid finding that is consistent with both clinical practice and the pathophysiology of BAD: BAD commonly causes secretory diarrhea due to the presence of bile acids in the colon. Importantly, the presence of SIBO did not impact symptomatic remission rates or the relationship between symptoms and endoscopic findings. The current study data in the overall cohort reinforce small post-hoc analyses of PREVENT trial subgroups (*n* = 24), which outlined a poor specificity (ie, high false positives) of endoscopic findings for CR (CDAI < 150).^8^ It is possible that BAD was a confounder heavily impacting these data.

Another aim of this study was to assess the relationship between Patient-Reported Outcome endpoints for CR and endoscopic inflammation. SF and AP have been identified as the 2 most important target endpoints when evaluating symptoms in CD for clinical trials.^19^ However, clinical trials have shown significant discordance between clinical and endoscopic results when using an SF threshold of 1 to define CR. Allowing patients to experience 1 bowel movement daily is a difficult, yet important target to strive for. Increasing the SF component of CR in trials has been needed to adjust for discordant symptom-based and endoscopic outcomes.^17^ However, experiencing 3 bowel movements daily is likely not ideal for patients. Our findings showed that BAD had a major impact on clinical scores even with the low SF threshold used, and in its presence, a vast majority will not achieve CR. This has a major implication for both clinical practice and trials. 7C4 is a routinely available test through multiple laboratories in the United States and can be obtained at the same time as routine blood tests. Objective BAD testing allows prompt treatment of BAD to eliminate potential symptom confounders, increases adherence to therapy, and can improve the quality of life.^12^

Study limitations include the generalizability of findings from a tertiary center. Although certain endoscopic and clinical scores were collected from chart review, clear documentation of validated endoscopic disease activity indices and/or absence of ulcers were obtained. Furthermore, a majority of SF and AP scores were prospectively assessed and documented in charts as these are commonly templated at our center. Additionally, analyses were unchanged in patients with prospectively collected scoring. There was a lower proportion of operated patients in the SIBO cohort, and this may bias the reported prevalence of SIBO and BAD in this study. Although certain patients were on antibiotics prior to SIBO testing, a large cohort without antibiotic use was analyzed with identical conclusions. A small minority of patients had a longer time between SIBO testing and clinical scoring. However, the mean time was 45 days and an analysis controlling for prospectively collected scores, antibiotic use, and clinical score within 30 days of the SIBO test was consistent with the results of the overall cohort. In addition, SIBO breath testing has highly variable sensitivity and specificity ranging from 17% to 93% and 30% to 86%, respectively, which may limit its consistency and validity.^20^ However, this further reinforces the importance of this study: The current testing for this entity in CD may be of limited utility.

## Conclusion

CD symptom–based and endoscopic scoring indices are discordant, and this discordance persists regardless of SIBO status. BAD is frequently observed in CD and has a major impact on CR scores and specifically differentiates those with elevated SF. Removing BAD as a confounder dramatically improves symptom–endoscopic concordance. This warrants further study for impacts on clinical trial outcomes. Clinicians should have a low threshold to test for serum 7C4 as a marker for BAD. Diagnosing the cause of symptoms is essential in IBD patients: In the presence of BAD, CD treatments will often not improve symptoms leading to reduced quality of life regardless of resolution of intestinal inflammation. BAD is likely a major source of untreated symptoms in CD.
